# From Health Determinant Benchmarks to Health Investment Benchmarks

**DOI:** 10.5888/pcd12.150010

**Published:** 2015-03-26

**Authors:** David Kindig

It is well established that the health system in the United States is underperforming. We lag most developed countries by a wide margin, despite spending more on health care (1). In addition, substantial geographic variation is seen in health outcomes in the United States, including unacceptable disparities in morbidity and mortality. Even absolute worsening of mortality rates in many US counties has been noted during the last several years (2).

Although we have better data systems than other countries have and a plethora of measurement efforts, these systems and efforts focus primarily on indicators or determinants of poor health, such as uninsured rates and smoking rates, or socioeconomic factors, such as income and education. This information on determinants — for health care and nonmedical determinants — is important, but it falls short: it does not tell us the level of financial investment needed to change each or all of the determinants and their resulting health outcomes. Population health improvement during the next decade requires a move from measuring only health determinants to developing investment benchmarks that can guide public and private action in communities and across the nation.

**Currently measured health outcomes and determinants.** For measuring broad population health, metrics such as *Healthy People 2020* (3) (for the nation), America’s Health Rankings (4) (for states), and County Health Rankings (5) (for counties) are useful for tracking progress and motivating change. In County Health Rankings, counties are ranked on an index of health outcomes and an index of the factors or determinants producing those outcomes. For each county in County Health Rankings, a “snapshot” provides national benchmarks for outcomes and determinants such as smoking rates and child poverty. These benchmarks are useful to local community public and private policy makers in choosing which priorities to tackle first.

**Moving to investment benchmarks.** Benchmarks for outcomes and determinants do not provide guidance on which new or additional population health investments are appropriate for a particular community. The poor performance of our health system compared with the health systems of other nations and the huge disparities in health outcomes in the United States must be the result of decades of varying levels of financial investment and health promotion in all policies. What guidance would trustees have for their decisions if a medium-sized community were to make available a pool of investment dollars, say $2 million per year for 10 years, from a public–private health outcome trust (6) or from reformed Internal Revenue Service community benefit policy aligned with the Community Health Needs Assessment process (7)? Why have we not added to our metrics reports useful benchmarks for optimal levels of per capita financial investment and policy strength across health-promoting factors to improve health determinants and ultimately outcomes?

It is largely because we have not yet made comparative effectiveness research on population health a high enough priority to enable us to know what the appropriate investment benchmarks would be. The Trust for America’s Health estimated in 2008 that investing $10 per person per year in proven community-based programs to increase physical activity, improve nutrition, and prevent smoking could save the United States more than $16 billion annually within 5 years (www.healthyamericans.org/reports/prevention08/Prevention08.pdf).

In 2009, Kim and Jennings found that more generous education spending, progressive tax systems, and more lenient welfare program rules at the state level help to improve population health (8). In a cross-national analysis, Bradley and colleagues (9) argued that an important reason for the poor performance of the US health system is the relative proportion of non–health care social services spending to health care services spending; in other developed countries it is 2.00 to 1, whereas in the United States it is 0.91 to 1.

**Challenges to population health investment benchmarks. **Why do we know so little? It is true that going beyond simply describing differences to finding causal pathways is complicated. Methods and data sets to explore these relationships are limited. In addition, we lack comparable investment information across small units of population, such as communities and counties. Tim Casper and I examined the availability of such data in a sample of Wisconsin counties for per capita expenditures in selected categories of health care, public health, human services, income support, job development, and education. We found that even this well-resourced state is challenged in locating usable data and having adequate information technology systems, and it lacks enterprise-wide coordination and geographic detail in data collection efforts (10).

An additional challenge is that communities vary in their outcomes and population health investments, so optimal additional population health investments would be different in different places. Consider, for example, 2 overall healthy states, Utah and North Dakota. In Utah, new population health investments might be directed toward education or health insurance, 2 areas in which Utah has relatively low rankings, whereas North Dakota might focus most effectively on health behavior issues, an area in which this state has fallen behind.

**Moving ahead.** Although it will not be easy, it is time to focus our analytic and policy attention on such population health investment benchmarks. We need much more evidence of the effectiveness of different programs and policies, particularly on cost-effectiveness beyond effectiveness itself. Such investment benchmarks would be helpful to guide work in the many places where community health improvement discussions are under way. Benchmarks need not be prescriptive, but they would be a menu of the investments likely to produce the best health outcome improvements. They would help ensure that local passion and commitment is channeled in an evidence-based direction while preserving autonomy and sensitivity to community preferences.

Some might argue that developing benchmarks is too difficult a task. However, we should not let the perfect be the enemy of the good. Although challenging, developing investment benchmarks is an essential step if we are to reverse the poor performance of our health system, with its negative impact on our quality of life and national productivity. It is time to get serious and use big data from public and private sources, such as electronic medical records and school records, and advanced modeling techniques to provide population health investment benchmarks to improve overall heath and reduce unacceptable health disparities across our wealthy country.
